# Healing Through Nutrition: Evaluating Dietary Support in Jordanian Hospitals

**DOI:** 10.3390/nu17040615

**Published:** 2025-02-08

**Authors:** Lana Alnimer, Razan Mahmoud Omoush, Amjad Al-Shalabi, Haitham Jahrami, Adam T. Amawi, Hadeel Ali Ghazzawi

**Affiliations:** 1 Department of Nutrition and Food Technology, School of Agriculture, The University of Jordan, Amman 11942, Jordan; l_nemer@ju.edu.jo (L.A.); razan.m.omoush@gmail.com (R.M.O.); amjo_shalabi09@yahoo.com (A.A.-S.); 2 Government Hospitals, Manama 329, Bahrain; haitham.jahrami@outlook.com; 3 Department of Psychiatry, College of Medicine and Health Sciences, Arabian Gulf University, Manama 329, Bahrain; 4 Department of Movement Sciences and Sports Training, School of Sport Sciences, The University of Jordan, Amman 11942, Jordan; a.amawi@ju.edu.jo

**Keywords:** hospital nutrition, macronutrients, micronutrients, dietary recommendations, Jordan

## Abstract

**Background/Objective:** Adequate nutrition is essential for patient recovery and overall health, yet hospital food services often fail to meet dietary guidelines. This study aimed to catch the gap between the dietary recommendation and the real intake. **Methods:** A total of 300 inpatients (100 per hospital type) were included in this cross-sectional study, which was conducted over two months. Nutritional intake was measured via weighed food records and actual intake was analyzed to calculate actual nutrient intake. Data were evaluated against dietary reference intakes (DRIs) and analyzed statistically via SPSS. One-way ANOVA and paired-sample *t* tests were used to identify significant differences between hospital categories and meal components. **Results:** The results revealed that private hospitals provided energy and macronutrient intakes closer to the recommended levels, with the total energy intake (2098.54 ± 97.33 kcal) exceeding the recommended level. Governmental and educational hospitals fell short, providing 1118.59 ± 68.21 kcal and 1285.91 ± 78.42 kcal, respectively. All hospital types served inadequate fiber, but private hospitals (23.18 ± 1.14 g) were closer to the recommendations. Micronutrient deficiencies were prevalent, particularly for vitamin D, vitamin E, and iron, across all hospital types. **Conclusions:** Nutritional intake varies significantly across Jordanian hospital categories, with private hospitals performing better than governmental and educational facilities do. Addressing these disparities through enhanced meal planning and monitoring is essential to improve patient health outcomes and reduce the risk of malnutrition.

## 1. Introduction

As a human right, good hospital nutrition means benefiting from the entire nutritional care process, which includes the government’s responsibility to ensure that patients have access to appropriate hospital diets—regular, therapeutic, or enteral. Nutritional treatment is a critical aspect of a patient’s overall care, starting with the identification of nutritional risks to prevent and treat malnutrition. This process involves offering a range of options from food to nutritional therapy. Nutritional therapy, a medical intervention, requires a medical indication and informed patient consent [1].

High-quality patient care involves both established standards of hospital care delivery, often supported by standardized order sets, and the implementation of quality assurance practices for continuous improvement. “Best practice” protocols, reviews, and recommendations [2] are crucial to ensure the consistent application of high standards, although their implementation can be inconsistent across hospitals. To address this, many hospitals have developed structured protocols and standardized order sets to guide patient care.

Hospital food services play a vital role in supporting patients’ well-being and recovery across all age groups. Assessing the adequacy of patients’ actual dietary intake is crucial for evaluating the acceptability of hospital meals, which can inform improvements in meal composition [3]. Monitoring nutritional intake is an integral part of best practices for managing hospitalized patients at nutritional risk [4]. If unaddressed during a patient’s hospital stay, malnutrition can lead to increased morbidity, prolonged hospitalization, and poorer survival outcomes [5].

This study aimed to address this gap by quantifying the daily nutrient intake of hospitalized patients over a 24-hour period in different types of hospitals, including a governmental (Al-Basheer Hospital), an educational (University of Jordan Hospital), and private hospitals (Al-Khaldi Hospital and Al-Isra’a Hospital). This research focused on identifying the actual nutrient intake in these hospitals and comparing the nutrient values provided with the Dietary Reference Intake (DRI) recommendations. Additionally, it examined the variation in the nutritional value of meals among governmental, educational, and private hospitals.

The study’s primary goal was to assess the adequacy of macronutrient and micronutrient intake in Jordanian hospitals (governmental, private, and educational) compared to dietary recommendations, aiming to identify gaps and enhance nutritional care and health outcomes. The findings provide valuable insights into the macro- and micronutrient adequacy of hospital meals and the nutritional status of inpatients. Ultimately, the results could guide interventions to improve nutritional care, reduce malnutrition, and minimize environmental and economic losses.

## 2. Materials and Methods

### 2.1. Design and Setting

This case series study was conducted between January and February 2020 in three hospitals in Jordan: a governmental hospital (Al-Basheer Hospital), an educational hospital (The University of Jordan Hospital), and two private hospitals (Al-Khaldi Hospital and Al-Isra’a Hospital). The sample size for this study was determined by selecting 300 in-patients across three types of hospitals in Jordan: governmental, private, and educational. Each hospital type contributed an equal number of participants, with 100 patients selected randomly from the inpatient lists during the two-month data collection period. This stratified approach ensured representation from each hospital category while maintaining a manageable and statistically analyzable sample size.

The formula for calculating sample size based on proportions is as follows:

$$n=(Z^{2}*p*(1-p))/e^{2}$$

Here,

*n*: Required sample size for each subgroup.
*Z*: *Z*-value corresponding to the desired confidence level (e.g., 1.96 for 95% confidence).
*p*: Expected proportion of the population with the characteristic of interest (e.g., 0.5 for maximum variability).
*e*: Margin of error (e.g., 0.05 for 5%).

For a confidence level of 95%, a margin of error of 5%, and assuming *p* = 0.5 (this is for the overall population),

$$n=({\left(1.96\right)}^{2}*0.5*(1-0.5))/{\left(0.05\right)}^{2}\approx 384$$

To ensure manageability, the study used a stratified approach, dividing the sample equally across three hospital types, resulting in 384\3 ≈ 128 participants per group.

The study adjusted this to a round number of 100 for feasibility.

### 2.2. Inclusion and Exclusion Criteria

Patients were eligible for inclusion if they were hospitalized during the survey period; had no impending medical tests, surgical procedures, or laboratory investigations; and had received hospital meals for at least one full day. Exclusion criteria applied to individuals under the age of 18, patients who were on nothing by mouth (NPO) orders, those undergoing cancer treatment, patients prescribed altered consistency diets (e.g., clear fluid, full fluid, or blenderized diets), or those receiving enteral or parenteral nutrition.

Ethical approval for the study was obtained (approval number 2020/20). The participants were provided with a detailed explanation of the study’s nature and objectives and were given the opportunity to ask questions. Written informed consent was obtained from all participants before enrollment. Confidentiality was strictly maintained, and the collected data were used solely to achieve the study’s objectives.

### 2.3. Nutritional Assessment

The nutritional demands for each patient were calculated via the Harris–Benedict equation (1918) to determine resting energy expenditure (REE), adjusted for factors such as height, weight, age, health status, comorbidities, physician recommendations, physical activity, and stress level [6]. Anthropometric measurements, including height and weight, were obtained from medical records at each hospital. Body mass index (BMI) was calculated as weight in kilograms divided by height in meters squared (kg/m²) and classified according to WHO standards [7].

### 2.4. Meal Preparation and Selection

To ensure unbiased results, all meals were served to participants following a standardized procedure, avoiding patient selection on the basis of personal preferences. Data on actual food intake were collected from three hospitals by weighing all meals served via an electronically calibrated balance (KERN & SOHN GmbH, Balingen, Germany, 0.01-gram sensitivity) at the time of service. Patient trays were labeled to track individual consumption, and after each meal—breakfast, lunch, and dinner—the remaining food was weighed to determine how much had been consumed. Actual intake was calculated as the difference between the amount of food served and the amount remaining after the meal. This process provided precise intake data across all hospital types [8].

Meals were served three times daily at fixed times: breakfast (7:00–8:00 AM), lunch (1:00–2:00 PM), and dinner (6:00–7:00 PM), with an average of 1200 kcal provided per day. The meal portions adhered to the Acceptable Macronutrient Distribution Range (AMDR): 45–65% carbohydrates, 20–35% fats, and 10–35% proteins [9]. Meals were based on scheduled hospital menus although some cooling occurred due to delays before serving. One to two hours after serving, food leftovers were collected, quantified, and subsequently discarded.

### 2.5. Comparison of Nutritional Demands, Served Food, and Actual Intake

The daily nutritional demands for each patient were recorded and compared to the energy provided in hospital meals, calculated via ESHA software Food Processor: 11.14.x (Released October 2023) and evaluated against the recommended dietary allowance (RDA) and dietary reference intake (DRI) guidelines. The ESHA (Elizabeth Stewart Hands and Associates (ESHA) Food Processor Nutrition Analysis Software) used in this study is widely recognized for its accuracy and reliability in calculating energy, macronutrients, and micronutrients. It is based on comprehensive food composition databases that include detailed nutrient profiles. While no software or database is entirely free from limitations, ESHA is considered one of the leading tools in this field and provides robust data for nutritional analysis. Carbohydrate intake was analyzed using the Dietary Reference Intakes (DRIs) and the acceptable macronutrient distribution range (AMDR) of 45–65% of total energy intake. This approach ensured alignment with international nutritional guidelines. Carbohydrate intake was evaluated against the acceptable macronutrient distribution range (AMDR) of 45–65% of total energy intake. Intakes exceeding 100 g per day were considered acceptable if they fell within this range.

Physicians’ recommendations were also considered on the basis of each patient’s health status.

To assess the actual intake, patient plates were weighed before and after meals across the three hospitals. Each plate was labeled with a unique code and the weights of individual food items were measured via a calibrated electronic balance (German, Kern, 0.01-gram sensitivity) at the time of service. After meals, labeled trays were collected and the remaining edible food waste was weighed. The average intake of each food item was calculated as the difference between the weight of the food served and the weight of the leftovers [8]. This procedure consistently applied to breakfast, lunch, and dinner for all the study participants.

### 2.6. Statistical Analysis

Data analysis was conducted via Statistical Package for the Social Sciences (SPSS) version 24. The results are expressed here as means ± standard deviations (SDs), with statistical significance set at *p* < 0.05. One-way analysis of variance (ANOVA), followed by the least significant difference (LSD) test, was used to evaluate significant differences between variable means across hospitals. Additionally, a paired-sample *t* test was applied to assess differences in nutrient content before and after meals.

## 3. Results

As shown in Table 1, approximately 53.3% of the participants were males, and the participants were distributed equally across the three types of hospitals—governmental, private, and educational—with one-third each type; approximately 36.2% of the participants were of normal weight, 39.3% were overweight and 15% were obese class 1 whereas much smaller percentages were found in the other BMI categories: 3.3% were underweight and 4% were obese class 2. Most of the participants were in the 51–70 years age group (43.3%), 32.7% of the participants were in the 31–50 years age group, 23.3% of the participants were in the 19–30 years age group, and only two participants were older than 70 years (1%). More than half of the participants were on a regular diet (54.7%) while 28.3% were on a diabetic diet.

Table 2 shows that the total energy consumption in governmental hospitals significantly (*p* < 0.05) differed from the recommendations for all age groups (*p* = 0.000). In private hospitals, the intake of 19-to-30-year-old patients was significantly greater (*p* = 0.029) than the recommended intake whereas there was no significant difference between the recommended intake and the actual intake for other age groups in private hospitals. All participants in the educational hospital consumed significantly (*p* < 0.05) less energy than recommended.

All age groups in both governmental hospitals and private hospitals consumed significantly more carbohydrates than the recommended group did (*p* < 0.05). In educational hospitals, the participants aged 31 years and above consumed significantly more carbohydrates than the recommended group did whereas those aged 30 years in educational hospitals consumed more carbohydrates than the recommended group did (*p* > 0.05).

Protein intake varied greatly between age groups and between hospitals and governmental hospitals; the 31-to 50-year-old age group significantly (*p* < 0.05) differed in protein intake from their recommended level whereas the other age groups did not. In private hospitals, all age groups consumed significantly more protein than recommended (*p* = 0.000). There was a significant difference in protein intake between the 31-to-50-year-old age group participants and their recommendations whereas there was no significant difference in protein intake between the intake group and the recommended group (*p* > 0.05).

Fat intake in governmental hospitals was significantly (*p* < 0.05) lower than the recommendations for all age groups. Fat intake in private hospitals was significantly (*p* < 0.05) greater than the recommendations for all age groups. However, there was no significant difference between the intake and recommendations of the participants in the educational hospital in terms of their fat intake.

Fiber intake in governmental hospitals was significantly (*p* < 0.05) lower than the recommendations for all age groups. Fiber intake in private hospitals was significantly (*p* < 0.05) lower than the recommended level for the 51–70 year age group and no significant difference was detected in the other age groups. While fiber intake in educational hospitals was also significantly (*p* < 0.05) lower than the recommendations for all age groups, except for the 14-to-18-year-old participants, there was no significant difference (*p* > 0.05) between their intake and their recommendations. Trans fat intake was substantially lower than the guideline in all hospitals, public, private, and educational, and for all age groups.

As stated in Table 3. There was no significant difference (*p* > 0.05) between the consumption of vitamin A and the recommendation for all age groups in the governmental hospital. Those aged 19–30 years and 31–50 years in private hospitals consumed significantly (*p* < 0.05) more vitamin A than did those in private hospitals. Participants in an educational hospital between the ages of 51 and 70 years consumed a significantly (*p* < 0.05) lower amount of vitamin A than the recommended amount did whereas other age groups in educational hospitals did not significantly differ between their intakes and their recommended amounts.

Unfortunately, the intakes of vitamin D and vitamin E for all participants in all age groups and all hospital types were significantly (*p* < 0.05) lower than the recommended values.

With respect to vitamin K in governmental hospitals, those aged 19–30 years and 51–70 years consumed significantly (*p* < 0.05) lower amounts of vitamin K than their recommendations did. In the 31-to-50-year-old age group, there was no significant difference between intake and the recommendations. The level of vitamin K in private hospitals was significantly (*p* < 0.05) greater than the recommended level in both the 19-to-30-year-old group and the 31-to-50-year-old group whereas there was no significant difference (*p* > 0.05) between the intake group and the recommended level. In the educational hospital, there was no significant difference (*p* > 0.05) between intake and the recommendation in all age groups except in the participants between 51 and 70 years old, who had significant differences (*p* < 0.05) between their intake and their recommendation.

Vitamin B1 intake in governmental and educational hospitals was significantly (*p* < 0.05) lower than the recommended level, but there was no significant difference (*p* > 0.05) between the intake level and the recommended level in private hospitals. The intake of vitamin B2 for all participants in all age groups and all hospital types was significantly (*p* < 0.05) lower than the recommended value, except in the 19-to-30-year-old participants, for whom there was no significant difference (*p* > 0.05) between their intake and their recommended value. Vitamin B3 consumption in governmental hospitals was significantly (*p* < 0.05) lower than the recommended level for all age groups. The consumption of vitamin K in private hospitals was statistically equal to the recommendations because no significant difference (*p* > 0.05) was detected, except in the age group of 51–70 years, where the intake was significantly lower than the recommendations. Additionally, vitamin B3 consumption in educational hospitals was significantly (*p* < 0.05) lower than the recommended level for all age groups.

Folic acid and vitamin B12 consumption in all hospital types and all age groups was significantly (*p* < 0.05) lower than the recommended levels. The consumption of vitamin C (ascorbic acid) in governmental hospitals was significantly (*p* < 0.05) lower than the recommended level for all age groups. In private hospitals, there was no significant difference (*p* > 0.05) between the intake of ascorbic acid and the recommendations in the 31–50 years and 51–70 years age groups whereas there was a significant difference (*p* < 0.05) between the intake and recommendation in the 19–30 years and 71-years-and-above age groups. The intake of vitamin C in educational hospitals was significantly lower than the recommendations for participants above 19 years of age.

As shown in Table 4. Calcium and iodine consumption levels in all hospital types and all age groups were significantly (*p* < 0.05) lower than the recommended levels. Iron intake was significantly (*p* < 0.05) lower than the recommended level in governmental hospitals. There was no significant difference (*p* > 0.05) between the intake level and the recommended level of iron in private hospitals whereas there was a significant difference between the intake level and the recommended level in educational hospitals within the age groups of 19–30 years and 31–50 years. Magnesium and potassium consumption in all hospital types and all age groups was significantly (*p* < 0.05) lower than the recommended levels. Sodium consumption in all hospital types and all age groups was significantly (*p* < 0.05) lower than the recommended value, except in participants aged 30 years and younger, for whom there was no significant difference (*p* > 0.05) between their intake and their recommended value in either governmental or educational hospitals. Almost all participants had phosphorus intakes that were significantly (*p* < 0.05) lower than the recommended level.

## 4. Discussion

Nutrient deficiencies have historically been examined within two distinct paradigms: one focusing on undernutrition, food insecurity, and micronutrient insufficiencies and the other addressing issues linked to overweight, obesity, and excessive dietary intake. These deficiencies play dual roles as both causes and consequences of illness, a relationship particularly pronounced among older adults. Notably, in England, the number of malnourished individuals discharged from NHS hospitals has increased 85% over a ten-year period [10].

Jordan’s healthcare sector is recognized for its high-quality and efficient services, solidifying its reputation as a regional medical hub [11]. A closer examination of three types of hospitals provides insights into the state of healthcare in Jordan while highlighting underlying socioeconomic disparities. Government hospitals predominantly serve low-to-middle-income families, educational hospitals cater to middle- and upper-middle-income groups, and private hospitals primarily serve high-income individuals.

The study population primarily comprised adults, with BMI data indicating that most participants were categorized as either of normal weight or overweight. Malnutrition rates were evenly distributed between males and females, indicating a comparable risk across genders. Most hospital visitors were aged 51–70 years, representing the most vulnerable group for malnutrition. Approximately half of the participants reported following a regular diet. Notably, in Jordan, as reflected in the sample, females tend to have higher BMI values than males do because of their body composition, which typically includes a greater proportion of adipose tissue [12].

An observed trend in the study indicated that the BMI increases with age, which contrasted with the findings of Wronka (2010), who reported an inverse relationship between age and the BMI irrespective of socioeconomic status [13]. This discrepancy can be attributed to the decline in physical activity that often accompanies aging, leading to weight gain and increased BMI values. A more sedentary lifestyle has been linked to reduced musculoskeletal function, further contributing to weight gain [14]. Additionally, the largest BMI group in private hospitals was the overweight category, highlighting another indication of socioeconomic disparities within Jordan’s population.

### 4.1. Malnutrition Among Different Types of Hospitals

The early detection of malnutrition in hospitalized patients is crucial for effective management and improved health outcomes [15]. Since inadequate food intake serves as a key indicator of malnutrition risk, addressing this issue through fortified therapeutic diets can help mitigate malnutrition [16]. Left untreated, overt malnutrition can lead to severe complications, including frequent infections, compromised immunity, and increased hospitalizations [17]. A study conducted by Baba et al. (1994) suggested that hospital malnutrition may be caused by either patients’ insufficient intake of food or nutrient deficiencies typically inherent in hospital diets [18].

Common factors contributing to hospital malnutrition include mealtime interruptions caused by medication rounds and other clinical activities, rigid food service practices, unfamiliar or unappealing food options, staffing and time shortages, and logistical issues such as the improper placement of food trays or packaging that is difficult for patients to open.

During hospitalization, patients are typically placed on therapeutic or standard diets by a registered dietician (RD) to manage nutrition-related disorders or to mitigate risks associated with acute and chronic adverse events arising from underlying medical conditions [19]. Adequate nutrition during hospitalization is a critical factor for patient recovery, making meal provision an integral component of medical treatment and nursing care [19] Research has reported that the prevalence of hospital malnutrition ranges from 56.2% to 62% [20].

Nutrient intake varies significantly across hospital types. Patients in private hospitals consume higher levels of total energy, carbohydrates, fats, proteins, and fiber than do those in educational and governmental hospitals. Similarly, the overall vitamin intake is better in private hospitals. While some participants exhibited carbohydrate intakes exceeding 100 g per day, these were within the acceptable macronutrient distribution range (45–65% of daily energy intake) and were not classified as deviations from the recommended intake. For example, vitamin A intake was highest in private hospitals, followed by government hospitals and then educational hospitals. Furthermore, the intakes of vitamins D, E, K, B1, and B2 were significantly greater in private hospitals than in educational and governmental facilities. Conversely, educational hospitals had better intakes of vitamin B12 and folic acid than government hospitals did. The higher intake of vitamin C in private hospitals may have reflected its higher cost, making it less accessible in other hospital settings. Furthermore, essential minerals also play a protective role against diseases, such as cancer, while deficiencies can lead to illness, and excessive intake may pose toxicity risks. Thus, it is crucial to adhere to daily mineral intake recommendations to maintain nutritional balance, particularly in hospitalized patients [21].

Food waste is considered a significant issue for prioritizing all hospital stakeholders responsible for food service operations. A study by Dumitriu et al. (2024) reported that approximately two-thirds of hospitalized patients were unsatisfied with the food served by the hospital in question whereas one-third of them were satisfied [22]. Unfortunately, this could result in an increased percentage of food waste among patients.

### 4.2. Comparing the Actual Intake with the Recommendations

Limited published data exist regarding hospital patient menus and the extent to which their nutrient contents align with recommended guidelines [20]. Our study corroborated this finding, revealing that private hospitals are the only facilities providing a nearly adequate macronutrient intake for patients. In contrast, educational and governmental hospitals generally fall short of recommendations for at least one nutrient or more.

Most patients across all hospital types consume nutrient amounts at or above the recommended levels, which may contribute to an increased body weight, an elevated BMI, and associated health issues. Notably, all the age groups in our study met or exceeded the protein requirements.

However, Simzari et al. (2017) reported that the average requirement of patients was estimated to be 2030.3 kcal/day whereas their actual intake was 1326 kcal/day. This may be explained by the overproduction of food during meal preparation in the kitchen, which may also contribute to food waste. In addition, more food waste is linked to a larger portion of the meal [23,24]. On the other hand, a low caloric intake and inadequate intake of protein, which have been noted in less than half of the meals reported by Curtis et al. (2018) and Dumitriu et al. (2024) [22,25], have been reported.

Private hospitals exceeded fat intake recommendations, educational hospitals provided fat intake close to the recommendations, and governmental hospitals offered fat amounts below the required levels. This disparity in fat intake may explain why patients in private hospitals tend to have higher body weights and BMI values. Similar results were reported by Moran et al. [26], who suggested that in all selected hospitals, most of the regular diets surpassed the recommended daily limits of the percentage of calories from fat and saturated fat and the milligrams of sodium. Compared with those of other nutrients, the dietary fiber, magnesium, and calcium contents were lower. Among the four hospitals, three hospitals provided higher amounts of total fat, saturated fat, and salt in accordance with the recommended intake. The long-term consumption of high-fat food could lead to considerable oxidative stress in tissues, especially brain tissues, and impaired mitochondrial function [27]. Additionally, it is broadly acknowledged that high fat consumption increases the risk of weight gain and an increased percentage of body fat [28].

Fiber intake fell below the recommended levels in all age groups in educational and governmental hospitals whereas in private hospitals, it generally met requirements across age groups, except in elderly individuals. This shortfall among elderly patients may be attributed to common challenges such as tooth loss and social isolation, which can reduce food intake. Insufficient fiber intake in this population can lead to gastrointestinal issues such as constipation and diarrhea, underscoring the importance of prioritizing fiber intake in hospital meal plans. Consistent with our results, Dener et al. (2022) assessed nutritional intake among hospitalized patients in four selected hospitals in Ankara and reported a lower intake of dietary fiber [29]. This may have been because the quantity of vegetables served was low, with the exception of starchy vegetables.

When menus are designed, it is essential to consider disease- or therapy-related feeding challenges, including a reduced appetite, altered taste perception, and difficulties with chewing or swallowing [30]. These factors must be addressed to ensure adequate nutrition and support patient recovery.

With respect to vitamin intake, vitamins A, K, B1, and C were provided in lower amounts in governmental hospitals and were most adequately served in private hospitals. However, the intake of vitamins D, E, B2, B3, folic acid, and B12 fell below the recommended levels across all age groups in all hospital types. These findings aligned with the results reported by El-Qudah (2018) [20].

Similarly, mineral intake was generally insufficient in all hospital types, with private hospitals offering relatively more adequate amounts than educational and governmental hospitals did. This underscores a broader trend of unmet dietary recommendations among hospitalized patients as highlighted in prior studies [20].

Patients across all age groups in all hospital types had significantly lower intakes of calcium, iodine, magnesium, potassium, sodium, and phosphorus than the recommended levels. Notably, patients in private hospitals exhibited iron intake that was statistically aligned with dietary recommendations. These findings aligned with those of El-Qudah (2018), who concluded that many hospitals fail to design regular diets that meet established dietary guidelines [20]. Moreover, Dener et al. (2022) assumed that potassium, magnesium, and dietary fiber intakes were lower as a result of very low quantities of green leafy vegetables served by hospitals. In fact, in the same study, among all four selected hospitals, the consumption of fruits was markedly low while starchy vegetable consumption reached 300% [29].

Despite the relative nutritional adequacy of almost all the diets of the hospitalized patients in Beirut, some patients consumed less than the RDA, as reported by Baba et al., (1994), which was consistent with our results [18]. Taking into account the palatability of food, food appearance, and specialized habits of patients for food preparations could contribute to the lower consumption of the foods served in hospitals. Low nutritional awareness among employees in the nutrition team was highlighted as a factor affecting the adequacy of hospitalized diets [31]. Besides the low staffing of nutritionists and inadequate facilities for parenteral and enteral nutrition (PN/EN) preparation, 50% of malnourished patients are not referred to nutritionists despite the critical role of nutrition intervention in improving nutritional status in hospitalized patients [32]. These are the major causes for inadequate hospital meals, aligning with findings in a Turkish study that found that nutritional risk patients are common in hospitals and often do not receive nutritional support when hospitalized [23]. Although comparing findings with studies from countries could add context, the differences in healthcare systems and nutritional practices between these regions and Jordan make such comparisons less relevant. This study focused on the unique nutritional challenges and healthcare practices in Jordan to provide recommendations that were specifically tailored to the local context and population needs.

## 5. Conclusions

This study revealed significant disparities in the nutritional quality of meals provided by hospitals in Jordan. While private hospitals delivered nutrient quantities closer to macronutrient guidelines and fulfilled some micronutrient requirements, governmental and educational hospitals consistently fell short of recommended levels. These deficiencies may adversely impact patient health and recovery, particularly for those already at nutritional risk. The findings underscore the need for improved dietary planning and monitoring across all hospital types to ensure adequate nutrient provision and to support patient health outcomes effectively.

## 6. Strengths

This study was the first to evaluate hospital nutrition in Jordan, providing important insights into nutritional gaps that affect patient recovery and health. The methodology was clear and reliable, ensuring strong results that highlighted critical issues in hospital meal planning. By addressing local challenges, the study offered practical recommendations to improve the quality of care and reduce the risks of complications caused by malnutrition. This research connects scientific findings with the needs of the healthcare system, making it a valuable step toward improving patient outcomes in Jordanian hospitals.

## 7. Limitations

This study had several limitations that should be considered when interpreting the results. First, the data collection period was limited to two months, which may not have captured seasonal variations in hospital meal planning and patient intake. Second, the sample size was confined to 300 inpatients, which, while providing valuable insights, might not have fully represented all hospitalized populations in Jordan. Third, self-reported data on patient food preferences and satisfaction were not included, which could have provided additional context on food intake and meal acceptability. Lastly, the study was restricted to three types of hospitals in Jordan, potentially limiting the generalizability of the findings to other countries or healthcare systems.

## 8. Future Suggestions

Future research should consider extending the duration of the study to capture seasonal variations in dietary intake and meal composition. Larger and more diverse samples, including a broader range of hospital settings and patient demographics, would enhance the generalizability of findings. Incorporating qualitative methods, such as patient satisfaction surveys or interviews, could provide deeper insights into barriers to adequate nutritional intake. Additionally, exploring the impact of specific interventions, such as menu modifications or staff training on nutrition, would help identify strategies to improve hospital food services and patient outcomes. Lastly, integrating biochemical markers could offer a more comprehensive assessment of the nutritional statuses of hospitalized patients.

## Figures and Tables

**Table 1:** Sociodemographic and anthropometric characteristics of the patients.

Variable	Classification	*n* (%)
**Gender**	Male	160 (53.3)
	Female	140 (46.7)
**Hospital Name**	Al-Basheer Hospital	100 (33.3)
	Khaldi Hospital	50 (16.7)
	Al-Esraa Hospital	50 (16.7)
	The Jordan University Hospital	100 (33.3)
**Hospital Category (Purpose)**	Governmental	100 (33.3)
	Private	100 (33.3)
	Educational	100 (33.3)
**BMI (kg/m^2^)**	Underweight	10 (3.3)
	Normal weight	109 (36.2)
	Overweight	118 (39.3)
	Obese class 1	45 (15.0)
	Obese class 2	12 (4.0)
	Obese class 3	6 (2.0)
**Age (year)**	19 to 30 years	70 (23.3)
	31 to 50 years	98 (32.7)
	51 to 70 years	130 (43.3)
	71 and more years	2 (0.7)
**Diet Type**	Regular	164 (54.7)
	Diabetic	85 (28.3)
	Low salt	28 (9.3)
	Others (Cardiac, Renal, Low-fat)	23 (7.7)

**Table 2:** Comparison of macronutrient intake (mean ± standard deviation (SD)) with recommendations (mean ± standard deviation (SD)) for different age groups and different hospital groups.

Hospital Category	Nutrients	Age Groups			
		19 to 30 Years	31 to 50 Years	51 to 70 Years	>71
		Mean ± SD			
		**Total Energy (g/day)**			
	**N**	24	30	46	-
**Governmental**	**Intake**	984.49 ± 114.26	1219.10 ± 136.98	1109.92 ± 103.55	-
	**Recom.**	1735.14 ± 48.74	1900.20 ± 67.53	1813.00 ± 43.60	-
	***p* value**	**0.000**	**0.000**	**0.000**	-
**Private**	**N**	27	33	38	2
	**Intake**	2468.52 ± 187.31	2189.31 ± 168.29	1780.29 ± 148.16	1653.47 ± 296.43
	**Recom.**	2037.07 ± 54.66	2033.83 ± 66.79	1818.49 ± 55.05	1726.86 ± 198.90
	***p* value**	**0.029**	0.369	0.810	0.589
**Educational**	**N**	19	35	46	-
	**Intake**	1300.59 ± 203.68	1389.37 ± 115.05	1218.56 ± 125.66	-
	**Recom.**	1893.13 ± 56.96	1925.84 ± 47.03	1755.09 ± 37.01	-
	***p* value**	**0.012**	**0.000**	**0.000**	-
		**Total Carbohydrate (g/day)**			
**Governmental**	**N**	24	30	46	-
	**Intake**	143.60 ± 15.48	165.34 ± 18.19	154.16 ± 13.44	-
	**Recom.**	100.00 ± 0.00	100.00 ± 0.00	100.00 ± 0.00	-
	***p* value**	**0.010**	**0.001**	**0.000**	-
**Private**	**N**	27	33	38	2
	**Intake**	341.30 ± 26.76	309.28 ± 26.82	241.91 ± 20.58	212.94 ± 61.86
	**Recom.**	100.00 ± 0.00	100.00 ± 0.00	100.00 ± 0.00	100.00 ± 0.00
	***p* value**	**0.000**	**0.000**	**0.000**	0.319
**Educational**	**N**	19	35	46	-
	**Intake**	153.18 ± 25.53	168.94 ± 17.67	157.20 ± 18.77	-
	**Recom.**	100.00 ± 0.00	100.00 ± 0.00	100.00 ± 0.00	-
	***p* value**	0.054	**0.000**	**0.004**	-
		**Total Protein (g/day)**			
**Governmental**	**N**	24	30	46	-
	**Intake**	50.55 ± 7.61	66.31 ± 7.94	58.73 ± 6.26	-
	**Recom.**	39.40 ± 1.45	48.62 ± 2.44	49.57 ± 1.36	-
	***p* value**	0.155	**0.031**	0.152	-
**Private**	**N**	27	33	38	2
	**Intake**	106.57 ± 6.37	94.25 ± 6.57	83.83 ± 6.43	79.34 ± 14.32
	**Recom.**	51.32 ± 2.06	54.11 ± 2.44	51.65 ± 2.05	53.79 ± 4.29
	***p* value**	**0.000**	**0.000**	**0.000**	0.238
**Educational**	**N**	19	35	46	-
	**Intake**	55.57 ± 8.29	64.22 ± 4.99	59.28 ± 5.87	-
	**Recom.**	44.88 ± 1.68	50.25 ± 1.60	48.62 ± 1.23	-
	***p* value**	0.23	**0.01**	0.06	-
		**Total Fat (g/day)**			
**Governmental**	**N**	24	30	46	-
	**Intake**	24.41 ± 3.50	33.87 ± 4.11	29.94 ± 3.06	-
	**Recom.**	38.56 ± 1.08	42.23 ± 1.50	40.29 ± 0.97	-
	***p* value**	**0.001**	**0.040**	**0.002**	-
**Private**	**N**	27	33	38	2
	**Intake**	79.66 ± 8.11	69.26 ± 6.54	57.88 ± 5.61	55.22 ± 0.26
	**Recom.**	45.27 ± 1.21	45.20 ± 1.48	40.41 ± 1.22	38.37 ± 4.42
	***p* value**	**0.000**	**0.001**	**0.004**	0.154
**Educational**	**N**	19	35	46	-
	**Intake**	52.71 ± 9.03	51.98 ± 4.31	40.43 ± 3.75	-
	**Recom.**	42.07 ± 1.27	42.80 ± 1.05	39.00 ± 0.82	-
	***p* value**	0.256	0.052	0.695	-
		**Fiber (g/day)**			
**Governmental**	**N**	24	30	46	-
	**Intake**	10.56 ± 0.91	13.51 ± 1.31	12.15 ± 0.92	-
	**Recom.**	27.50 ± 0.00	27.50 ± 0.00	27.50 ± 0.00	-
	***p* value**	**0.000**	**0.000**	**0.000**	-
**Private**	**N**	27	33	38	2
	**Intake**	24.67 ± 1.92	24.24 ± 2.20	21.74 ± 1.83	12.87 ± 3.70
	**Recom.**	27.50 ± 0.00	27.50 ± 0.00	27.50 ± 0.00	27.50 ± 0.00
	***p* value**	0.153	0.147	**0.003**	0.157
**Educational**	**N**	19	35	46	-
	**Intake**	11.91 ± 1.87	12.03 ± 1.03	10.97 ± 1.10	-
	**Recom.**	27.50 ± 0.00	27.50 ± 0.00	27.50 ± 0.00	-
	***p* value**	**0.000**	**0.000**	**0.000**	-
		**Trans Fat (mg/day)**			
**Governmental**	**N**	24	30	46	-
	**Intake**	0.06 ± 0.02	0.07 ± 0.02	0.04 ± 0.01	-
	**Recom.**	1.93 ± 0.05	2.11 ± 0.08	2.01 ± 0.05	-
	***p* value**	**0.000**	**0.000**	**0.000**	-
**Private**	**N**	27	33	38	2
	**Intake**	0.19 ± 0.06	0.10 ± 0.03	0.09 ± 0.02	0.06 ± 0.06
	**Recom.**	2.26 ± 0.06	2.26 ± 0.07	2.02 ± 0.06	1.92 ± 0.22
	***p* value**	**0.000**	**0.000**	**0.000**	**0.000**
**Educational**	**N**	19	35	46	-
	**Intake**	0.01 ± 0.005	0.01 ± 0.003	0.001 ± 0.001	-
	**Recom.**	2.10 ± 0.06	2.14 ± 0.05	1.95 ± 0.04	-
	***p* value**	**0.000**	**0.000**	**0.000**	-

**Table 3:** Comparison of vitamin intake with recommendations for different age groups and different hospital groups.

Hospital Category	Nutrients	Age Groups			
		19 to 30 Years	31 to 50 Years	51 to 70 Years	71 and More Years
		Mean ± SD			
		**Vitamin A (IU/day)**			
**Governmental**	**N**	24	30	46	-
	**Intake**	2720.86 ± 687.61	3801.28 ± 631.57	3536.48 ± 595.56	-
	**Recom.**	2536.23 ± 65.40	2755.55 ± 59.66	2826.09 ± 43.64	-
	***p* value**	0.795	0.110	0.243	-
**Private**	**N**	27	33	38	2
	**Intake**	5926.46 ± 1348.06	5603.84 ± 992.93	3503.21 ± 777.77	952.51 ± 48.09
	**Recom.**	2679.01 ± 65.33	2696.97 ± 58.68	2701.75 ± 54.50	2666.67 ± 333.34
	***p* value**	**0.022**	**0.005**	0.308	0.139
**Educational**	**N**	19	35	46	-
	**Intake**	2144.57 ± 483.81	2269.81 ± 346.16	1748.08 ± 258.77	-
	**Recom.**	2607.84 ± 82.03	2676.19 ± 57.14	2637.68 ± 49.50	-
	***p* value**	0.349	0.237	**0.002**	-
		**Vitamin D (IU/day)**			
**Governmental**	**N**	24	30	46	-
	**Intake**	0.86 ± 0.54	0.86 ± 0.43	0.30 ± 0.21	-
	**Recom.**	600.00 ± 0.00	600.00 ± 0.00	600.00 ± 0.00	-
	***p* value**	**0.000**	**0.000**	**0.000**	-
**Private**	**N**	27	33	38	2
	**Intake**	8.55 ± 3.67	3.98 ± 1.47	3.39 ± 1.62	1.50 ±1.50
	**Recom.**	600.00 ± 0.00	600.00 ± 0.00	600.00 ± 0.00	800.00 ± 100.00
	***p* value**	**0.000**	**0.000**	**0.000**	0.092
**Educational**	**N**	19	35	46	-
	**Intake**	0.05 ± 0.05	0.28 ± 0.13	0.26 ± 0.10	-
	**Recom.**	600.00 ± 0.00	600.00 ± 0.00	600.00 ± 0.00	-
	***p* value**	**0.000**	**0.000**	**0.000**	-
		**Vitamin E (IU/day)**			
**Governmental**	**N**	24	30	46	-
	**Intake**	2.39 ± 0.38	3.28 ± 0.41	3.14 ± 0.32	-
	**Recom.**	22.39 ± 0.00	22.39 ± 0.00	22.39 ± 0.00	-
	***p* value**	**0.000**	**0.000**	**0.000**	-
**Private**	**N**	27	33	38	2
	**Intake**	5.59 ± 0.71	4.71 ± 0.91	3.50 ± 0.40	1.21 ± 0.30
	**Recom.**	22.39 ± 0.00	22.39 ± 0.00	22.39 ± 0.00	22.39 ± 0.00
	***p* value**	**0.000**	**0.000**	**0.000**	**0.009**
**Educational**	**N**	19	35	46	-
	**Intake**	2.42 ± 0.51	2.55 ± 0.30	2.05 ± 0.26	-
	**Recom.**	22.39 ± 0.00	22.39 ± 0.00	22.39 ± 0.00	-
	***p* value**	**0.000**	**0.000**	**0.000**	-
		**Vitamin K (mcg/day)**			
**Governmental**	**N**	24	30	46	-
	**Intake**	31.73 ± 15.67	94.79 ± 20.00	70.39 ± 14.62	-
	**Recom.**	99.13 ± 2.94	109.00 ± 2.68	112.17 ± 1.96	-
	***p* value**	**0.000**	0.481	**0.006**	-
**Private**	**N**	27	33	38	2
	**Intake**	177.21 ± 20.26	172.87 ± 27.59	133.29 ± 17.96	34.68 ± 25.69
	**Recom.**	105.56 ± 2.94	106.36 ± 2.64	106.58 ± 2.45	105.00 ± 15.00
	***p* value**	**0.001**	**0.019**	0.137	0.096
**Educational**	**N**	19	35	46	-
	**Intake**	89.39 ± 22.11	82.90 ± 13.95	68.67 ± 11.69	-
	**Recom.**	102.35 ± 3.69	105.43 ± 2.57	103.70 ± 2.23	-
	***p* value**	0.561	0.117	**0.003**	-
		**Vitamin B1 Thiamin (mg/day)**			
**Governmental**	**N**	24	30	46	-
	**Intake**	0.44 ± 0.77	0.58 ± 0.08	0.53 ± 0.06	-
	**Recom.**	1.13 ± 0.01	1.16 ± 0.01	1.17 ± 0.01	-
	***p* value**	**0.000**	**0.000**	**0.000**	-
**Private**	**N**	27	33	38	2
	**Intake**	1.41 ± 0.17	1.25 ± 0.15	1.04 ± 0.11	0.98 ± 0.29
	**Recom.**	1.15 ± 0.01	1.15 ± 0.01	1.16 ± 0.01	1.15 ± 0.05
	***p* value**	0.124	0.528	0.328	0.608
**Educational**	**N**	19	35	46	-
	**Intake**	0.83 ± 0.14	0.89 ± 0.11	0.79 ± 0.11	-
	**Recom.**	1.14 ± 0.01	1.15 ± 0.01	1.15 ± 0.01	-
	***p* value**	0.056	**0.026**	**0.003**	-
		**Vitamin B2 Riboflavin (mg/day)**			
**Governmental**	**N**	24	30	46	-
	**Intake**	0.31 ± 0.05	0.41 ± 0.05	0.38 ± 0.04	-
	**Recom.**	1.16 ± 0.02	1.22 ± 0.02	1.25 ± 0.01	-
	***p* value**	**0.000**	**0.000**	**0.000**	-
**Private**	**N**	27	33	38	2
	**Intake**	1.00 ± 0.11	0.84 ± 0.09	0.72 ± 0.08	0.64 ± 0.06
	**Recom.**	1.20 ± 0.02	1.21 ± 0.02	1.21 ± 0.02	1.20 ± 0.10
	***p* value**	0.053	**0.000**	**0.000**	**0.045**
**Educational**	**N**	19	35	46	-
	**Intake**	0.65 ± 0.10	0.73 ± 0.07	0.64 ± 0.08	-
	**Recom.**	1.18 ± 0.02	1.20 ± 0.02	1.19 ± 0.01	-
	***p* value**	**0.000**	**0.000**	**0.000**	-
		**Vitamin B3 Niacin (mg/day)**			
**Governmental**	**N**	24	30	46	-
	**Intake**	4.17 ± 0.68	5.63 ± 0.78	5.06 ± 0.59	-
	**Recom.**	14.61 ± 0.20	15.27 ± 0.18	15.48 ± 0.13	-
	***p* value**	**0.000**	**0.000**	**0.000**	-
**Private**	**N**	27	33	38	2
	**Intake**	14.30 ± 2.25	12.43 ± 1.39	10.36 ± 1.32	9.70 ± 0.06
	**Recom.**	15.04 ± 0.20	15.09 ±0.18	15.11 ± 0.16	15.00 ± 1.00
	***p* value**	0.745	0.063	**0.001**	0.112
**Educational**	**N**	19	35	46	-
	**Intake**	8.96 ± 1.62	11.24 ± 1.38	10.16 ± 1.40	-
	**Recom.**	14.82 ± 0.25	15.02 ± 0.17	14.91 ± 0.15	-
	***p* value**	**0.002**	**0.009**	**0.001**	-
		**Vitamin B9 Folic Acid (mcg/day)**			
**Governmental**	**N**	24	30	46	-
	**Intake**	45.64 ± 9.37	54.43 ± 9.10	53.97 ± 7.96	-
	**Recom.**	400.00 ± 0.00	400.00 ± 0.00	400.00 ± 0.00	-
	***p* value**	**0.000**	**0.000**	**0.000**	-
**Private**	**N**	27	33	38	2
	**Intake**	116.57 ± 18.33	90.99 ± 17.60	63.98 ± 12.21	86.32 ± 19.09
	**Recom.**	400.00 ± 0.00	400.00 ± 0.00	400.00 ± 0.00	400.00 ± 0.00
	***p* value**	**0.000**	**0.000**	**0.000**	**0.039**
**Educational**	**N**	19	35	46	-
	**Intake**	96.41 ± 17.45	97.35 ± 12.24	78.41 ± 11.41	-
	**Recom.**	400.00 ± 0.00	400.00 ± 0.00	400.00 ± 0.00	-
	***p* value**	**0.000**	**0.000**	**0.000**	-
		**Vitamin B12 Cobalamin (mcg/day)**			
**Governmental**	**N**	24	30	46	-
	**Intake**	0.02 ± 0.01	0.05 ± 0.02	0.04 ± 0.01	-
	**Recom.**	2.40 ± 0.00	2.40 ± 0.00	2.40 ± 0.00	-
	***p* value**	**0.000**	**0.000**	**0.000**	-
**Private**	**N**	27	33	38	2
	**Intake**	0.79 ± 0.23	0.60 ± 0.18	0.68 ± 0.21	0.01 ± 0.01
	**Recom.**	2.40 ± 0.00	2.40 ± 0.00	2.40 ± 0.00	2.40 ± 0.00
	***p* value**	**0.000**	**0.000**	**0.000**	**0.001**
**Educational**	**N**	19	35	46	-
	**Intake**	0.58 ± 0.18	0.95 ± 0.14	0.86 ± 0.12	-
	**Recom.**	2.40 ± 0.00	2.40 ± 0.00	2.40 ± 0.00	-
	***p* value**	**0.000**	**0.000**	**0.000**	-
		**Vitamin C Ascorbic Acid (mg/day)**			
**Governmental**	**N**	24	30	46	-
	**Intake**	50.35 ± 81.66	64.89 ± 7.00	49.96 ± 4.93	-
	**Recom.**	79.56 ± 1.47	83.50 ± 1.34	86.09 ± 0.98	-
	***p* value**	**0.005**	**0.007**	**0.000**	-
**Private**	**N**	27	33	38	2
	**Intake**	109.61 ± 15.32	85.92 ± 10.78	69.36 ± 9.57	36.23 ± 6.60
	**Recom.**	82.78 ± 1.47	83.18 ± 1.32	83.29 ± 1.23	82.50 ± 7.50
	***p* value**	**0.09**	0.798	0.145	**0.012**
**Educational**	**N**	19	35	46	-
	**Intake**	37.12 ± 6.15	39.77 ± 3.82	37.14 ± 4.38	-
	**Recom.**	81.18 ± 1.85	82.71 ± 1.29	81.85 ± 1.11	-
	***p* value**	**0.000**	**0.000**	**0.000**	-

**Table 4:** Comparison of mineral intake with recommendations for different age groups and different hospital groups.

Hospital Category	Nutrients	Age Groups			
		19 to 30 Years	31 to 50 Years	51 to 70 Years	71 and More Years
		Mean ± SD			
		**Calcium (mg/day)**			
**Governmental**	**N**	24	30	46	-
	**Intake**	377.83 ± 40.10	397.82 ± 37.65	359.39 ± 33.86	-
	**Recom.**	1000.00 ± 0.00	1000.00 ± 0.00	1052.17 ± 13.09	-
	***p* value**	**0.000**	**0.000**	**0.000**	-
**Private**	**N**	27	33	38	2
	**Intake**	715.95 ± 58.48	643.91 ± 52.73	541.50 ± 40.02	502.49 ± 102.85
	**Recom.**	1000.00 ± 0.00	1000.00 ± 0.00	1089.47 ± 16.35	1200.00 ± 0.00
	***p* value**	**0.000**	**0.000**	**0.000**	0.093
**Educational**	**N**	19	35	46	-
	**Intake**	653.77 ± 85.14	686.75 ± 40.11	569.88 ± 46.09	-
	**Recom.**	1000.00 ± 0.00	1000.00 ± 0.00	1108.70 ± 14.85	-
	***p* value**	**0.001**	**0.000**	**0.000**	-
		**Iodine (mcg/day)**			
**Governmental**	**N**	24	30	46	-
	**Intake**	3.18 ± 1.13	2.23 ± 0.70	2.28 ± 0.66	-
	**Recom.**	150.00 ± 0.00	150.00 ± 0.00	150.00 ± 0.00	-
	***p* value**	**0.000**	**0.000**	**0.000**	-
**Private**	**N**	27	33	38	2
	**Intake**	9.53 ± 7.95	1.20 ± 0.71	1.07 ± 0.60	0.00 ± 0.00
	**Recom.**	150.00 ± 0.00	150.00 ± 0.00	150.00 ± 0.00	150.00 ± 0.00
	***p* value**	**0.000**	**0.000**	**0.000**	**0.000**
**Educational**	**N**	19	35	46	-
	**Intake**	0.001 ± 0.004	0.002 ± 0.002	0.003 ± 0.002	-
	**Recom.**	150.00 ± 0.00	150.00 ± 0.00	150.00 ± 0.00	-
	***p* value**	**0.000**	**0.000**	**0.000**	-
		**Iron (mg/day)**			
**Governmental**	**N**	24	30	46	-
	**Intake**	3.92 ± 0.58	5.65 ± 0.68	4.63 ± 0.46	-
	**Recom.**	14.96 ± 0.98	11.67 ± 0.89	8.00 ± 0.00	-
	***p* value**	**0.000**	**0.000**	**0.000**	-
**Private**	**N**	27	33	38	2
	**Intake**	13.52 ± 1.22	12.58 ± 1.37	10.91 ± 1.02	6.85 ± 0.43
	**Recom.**	12.81 ± 0.98	12.55 ± 0.88	8.00 ± 0.00	8.00 ± 0.00
	***p* value**	0.708	0.985	**0.007**	0.224
**Educational**	**N**	19	35	46	-
	**Intake**	7.94 ± 1.29	8.18 ± 0.78	7.02 ± 0.78	-
	**Recom.**	13.88 ± 1.23	12.86 ± 0.86	8.00 ± 0.00	-
	***p* value**	**0.004**	**0.001**	0.215	-
		**Magnesium (mg/day)**			
**Governmental**	**N**	24	30	46	-
	**Intake**	59.56 ± 7.90	70.87 ± 9.03	56.91 ± 6.27	-
	**Recom.**	337.39 ± 8.83	383.33 ± 8.95	393.91 ± 6.55	-
	***p* value**	**0.000**	**0.000**	**0.000**	-
**Private**	**N**	27	33	38	2
	**Intake**	172.50 ± 15.66	157.22 ± 16.11	150.52 ± 16.39	67.97 ± 28.50
	**Recom.**	356.67 ± 8.82	374.55 ± 8.80	375.26 ± 8.17	370.00 ± 50.00
	***p* value**	**0.000**	**0.000**	**0.000**	**0.045**
**Educational**	**N**	19	35	46	-
	**Intake**	70.81 ± 11.98	83.39 ± 9.47	76.32 ± 9.84	-
	**Recom.**	347.06 ± 11.07	371.43 ± 8.57	365.65 ± 7.43	-
	***p* value**	**0.000**	**0.000**	**0.000**	-
		**Potassium (mg/day)**			
**Governmental**	**N**	24	30	46	-
	**Intake**	658.52 ± 66.12	864.63 ± 80.18	766.99 ± 64.02	-
	**Recom.**	4700.00 ± 0.00	4700.00 ± 0.00	4700.00 ± 0.00	-
	***p* value**	**0.000**	**0.000**	**0.000**	-
**Private**	**N**	27	33	38	2
	**Intake**	1845.06 ± 179.82	1733.48 ± 169.40	1585.30 ± 141.34	890.35 ± 7.35
	**Recom.**	4700.00 ± 0.00	4700.00 ± 0.00	4700.00 ± 0.00	4700.00 ± 0.00
	***p* value**	**0.000**	**0.000**	**0.000**	**0.001**
**Educational**	**N**	19	35	46	-
	**Intake**	600.63 ± 105.55	771.76 ± 83.40	710.26 ± 86.15	-
	**Recom.**	4700.00 ± 0.00	4700.00 ± 0.00	4700.00 ± 0.00	-
	***p* value**	**0.000**	**0.000**	**0.000**	-
		**Sodium (mg/day)**			
**Governmental**	**N**	24	30	46	-
	**Intake**	2064.13 ± 356.13	3427.95 ± 429.19	2946.93 ± 322.53	-
	**Recom.**	1500.00 ± 0.00	1500.00 ± 0.00	1300.00 ± 0.00	-
	***p* value**	0.127	**0.000**	**0.000**	-
**Private**	**N**	27	33	38	2
	**Intake**	8254.33 ± 1203.13	7443.72 ± 1008.31	4996.59 ± 550.60	3374.60 ± 1410.02
	**Recom.**	1500.00 ± 0.00	1500.00 ± 0.00	1300.00 ± 0.00	1200.00 ± 0.00
	***p* value**	**0.000**	**0.000**	**0.000**	0.366
**Educational**	**N**	19	35	46	-
	**Intake**	2493.89 ± 540.51	2608.64 ± 341.99	2454.16 ± 349.83	-
	**Recom.**	1500.00 ± 0.00	1500.00 ± 0.00	1300.00 ± 0.00	-
	***p* value**	0.085	**0.003**	**0.002**	-
		**Phosphorus (mg/day)**			
**Governmental**	**N**	24	30	46	-
	**Intake**	167.72 ± 26.34	264.30 ± 31.47	207.42 ± 21.37	-
	**Recom.**	700.00 ± 0.00	700.00 ± 0.00	700.00 ± 0.00	-
	***p* value**	**0.000**	**0.000**	**0.000**	-
**Private**	**N**	27	33	38	2
	**Intake**	629.11 ± 69.05	538.51 ± 50.07	437.26 ± 43.41	452.37 ± 54.84
	**Recom.**	700.00 ± 0.00	700.00 ± 0.00	700.00 ± 0.00	700.00 ± 0.00
	***p* value**	0.314	**0.003**	**0.000**	0.139
**Educational**	**N**	19	35	46	-
	**Intake**	254.04 ± 40.73	294.86 ± 30.45	265.86 ± 31.07	-
	**Recom.**	700.00 ± 0.00	700.00 ± 0.00	700.00 ± 0.00	-
	***p* value**	**0.000**	**0.000**	**0.000**	-

## Data Availability

The data presented in this study are available upon request from the corresponding authors.

## Abbreviations

The following abbreviations are used in this manuscript:

RDA	Recommended daily allowance
DRIs	Dietary Reference Intakes
BMI	Body Mass Index
WHO	World health organization
RD	registered dietitian
ANOVA	One-way Analysis of Variance
LSD	Least Significant Difference
SD	standard deviation
SPSS	Statistical Package for the Social Sciences
REE	resting energy expenditure
NPO	Nothing per mouth
